# Time spent playing outdoors after school and its relationship with independent mobility: a cross-sectional survey of children aged 10–12 years in Sydney, Australia

**DOI:** 10.1186/1479-5868-6-15

**Published:** 2009-03-16

**Authors:** Li Ming Wen, James Kite, Dafna Merom, Chris Rissel

**Affiliations:** 1 Health Promotion Service, Sydney South West Area Health Service, Syndey, Australia; 2 School of Public Health, University of Sydney, Sydney, Australia

## Abstract

**Background:**

Time spent outdoors is positively associated with physical activity and has been suggested as a proxy for physical activity of children. The role of children's independence in physical activity and time spent outdoors is less understood. This study aimed to assess how much time children spent playing outdoors after school, and to explore the relationship between outdoor play and independence among children aged 10–12 years.

**Method:**

Children recorded how much time they spent playing outdoors or watching TV/videos or playing computer games after school using a five-day diary, and also reported whether they were allowed to walk on their own in their neighbourhood as an indicator of their independent mobility. Parents were surveyed on family demographics and perception of neighbourhood safety. The surveys were conducted in late 2006 as part of the Central Sydney Walk to School program which involved 1975 children and their parents from 24 primary schools. Factors associated with time spent playing outdoors were determined by logistic regression modelling.

**Results:**

Thirty-seven per cent of children spent less than half an hour a day playing outdoors after school, and 43% spent more than 2 hours a day watching TV, videos or playing computer games. Forty-eight per cent of children were allowed to walk on their own near where they lived. Children's independent mobility was significantly associated with outdoor play after adjusting for other confounders. Compared with those who were never allowed to walk on their own near where they lived, students who were allowed to walk on their own were significantly more likely to spend more than half an hour a day playing outdoors after school with an adjusted odds ratio of 2.6, 95% CI 1.84–3.58, P < 0.001.

**Conclusion:**

The findings that a significant proportion of children spend less than half an hour a day playing outdoors after school and have excessive screen time have important implications for physical activity promotion and obesity prevention. The study also suggests that children's independent mobility should be considered in research and evaluation into children's play and physical activity. Environments that promote greater independent mobility in children may increase their physical activity levels and hence reduce their risk of overweight/obesity.

## Background

Promoting physical activity is an important strategy in tackling the epidemic of childhood overweight and obesity. Children participating in leisure-time physical activity have been found to be significantly less likely to be overweight than children who reported no leisure-time physical activity [1,2]. Australian physical activity guidelines recommend children spend a minimum of 60 minutes per day in moderate to vigorous physical activity, and be part of play, games, sports, transportation, recreation, physical education class or planned exercise [3]. Conversely, they should spend no more than 120 minutes per day engaged in small screen entertainment [3].

However, assessing children's level of physical activity can be challenging and costly. To our knowledge, to date there are no validated survey questionnaires that have been developed for assessing level of physical activity for primary school children. Direct measures such as observation and accelerometers can be used but are often not feasible in large epidemiologic studies.

Time spent outdoors is positively associated with physical activity levels of children [4-6], and has been suggested as a proxy for physical activity [7]. A recent study conducted by Cleland et al concluded that encouraging 10- to 12-year-old children to spend more time outdoors may be an effective strategy for increasing physical activity and preventing overweight and obesity in children [8].

Children's independence can be defined as the ability to take responsibility for their own behaviour without unnecessary reliance on their parents or carers. Every child has the potential for some degree of independence in their daily lives. Developing independence is an important goal for all children and is an ongoing process that continues throughout childhood, adolescence, and even into adulthood [9]. However, the role of children's independence in physical activity and time spent outdoors is less understood.

The aims of this study were to assess how much time students spent playing outdoors after school, and to explore the relationship between outdoor play and independent mobility among children aged 10 – 12 years (5^th ^and 6^th ^year students).

## Methods

### Study design

A cross-sectional survey was conducted in October, 2006 as part of the evaluation of the Central Sydney Walk to School Research Program, which has been reported in detail elsewhere [10]. The research program was approved by the ethics committees of Sydney South West Area Health Service, and the NSW Department of Education and Training.

### Study participants

Twenty-four schools located in inner west Sydney volunteered to be part of the study. Participating schools varied in size, socio-economic status of students and their families, and cultural mix. All 5^th ^and 6^th ^year students, aged 10–12 years, in the participating schools (n = 2230) were recruited for the survey, and their parents were also invited to take part in the survey with a letter giving detailed information about the study.

### Data collection

The student survey was conducted in class and supervised by teachers over five consecutive school days, using a diary format. Class teachers distributed copies of the parent survey to their students to take home. Parents completed the surveys at home, if they chose to participate, and returned their completed survey to the class teacher. The student and parent surveys were matched through a previously used anonymous record linkage technique [11] based on the first letter of surname, class and year, and birth date of student.

### Measures

Time spent playing outdoors, time spent on watching TV/videos or playing computer games (also known as "screen time") and the independent mobility were collected from students' surveys. Family and demographic information was collected from the parents.

#### ➢ Time spent playing outdoors and screen time

Student surveys contained questions that asked students to recall how much time they spent playing outdoors after school yesterday. Students answered the question by selecting one time slot out of six incremental options ranging from none to more than three hours with intervals of half an hour. The data were then summed and averaged over five days. An average time per day spent playing outdoors after school and an average screen time were calculated. Time spent playing outdoors for less than half an hour a day was considered "low". This cut-point was used because it represents a very low amount of physical activity and as such, this group was at greatest risk of developing health-related problems. Similarly, daily screen time (i.e. watching TV/videos and playing computer games) was also calculated by summing the responses for these activities and averaged over five days.

#### ➢ Independent Mobility

Independent mobility was assessed by a single question, "are you allowed to walk on your own, near where you live?" Children were able to select one of three options, "mostly", "sometimes" and "never".

#### ➢ Family and demographic information and neighbourhood safety

The information was collected in the parent survey, which included their relation to the child, age, education level, employment status, language spoken at home, and the number of children at home. Parents were also asked if they agreed or disagreed with the statement, "I live in a safe neighbourhood" by a scale from "1" to '5" where "1" represents "strongly agreed" and "5" represents "strongly disagreed".

### Analysis

Statistical analyses were carried out using the computer package SPSS Complex Samples Statistics (Version 14.0) [12], which incorporates the sampling design into survey analysis. It is widely used to allow for cluster sampling and unequal probability of selection.

Relationships between study and outcome variables were examined using Pearson chi-square tests and Mantel-Haesnszel chi-square tests for trend in proportions. Variables that were found to be associated with time spent playing outdoors on bi-variate analyses were entered into a logistic regression model.

In the logistic regression analysis all variables were entered in one step and removed from the model according to their statistical significance on entry, and whether they met the removal criterion (P = 0.10). Adjusted odds ratios (AORs) with 95% confidence intervals were calculated as a measure of association. A similar process was applied to examine whether correlates were different for boys or girls.

## Results

### Response rate and study respondents

A total of 1974 students completed a five-day diary survey giving a response rate of 89%, but only 1362 parents completed the parent survey giving a response rate of 61%. In this analysis, children without their parent survey data were excluded (n = 612). There were no statistically significant differences in relation to time spent playing outdoors, screen time and the independent mobility between those who remained in the analysis and those excluded.

Table 1 shows the characteristics of the students and parents remaining in the analysis (n = 1362). Of these students, 47% were boys. The majority of survey respondents were mothers (80%). Forty per cent of parents were employed full-time, 42% had a tertiary degree, and 42% spoke a language other than English at home. About half of the families had two children at home.

**Table 1:** Characteristics of the study participants

**Characteristics**	**N = 1362** n* (%)
**Student Gender**	
Girl	710 (53%)
Boy	638 (47%)

**Student School Year**	
Year 5	693 (51%)
Year 6	656 (49%)

**Respondent**	
Mother	1061 (80%)
Father	241 (18%)
Other	28 (2%)

**Parent Age**	
Less than 30 yrs	33 (3%)
30–39 yrs	406 (30%)
40–49 yrs	777 (58%)
50+	128 (9%)

**Language spoken at home**	
English	792 (58%)
Other	570 (42%)

**Parent Education**	
Primary and some high school	128 (12%)
Completed high school	245 (22%)
Technical certificate or diploma	277 (25%)
University/Other tertiary degree	473 (42%)

**Parent Employment Status**	
Employed full-time	535 (40%)
Employed part-time	378 (28%)
Full/part-time student	38 (3%)
Home duties	264 (20%)
Unemployed	59 (4%)
Retired/other	17 (5%)

**Number of Children at home**	
1	336 (25%)
2	643 (48%)
3	277 (21%)
4+	77 (6%)

**Parent Living with Partner**	
Yes	955 (84%)
No	185 (16%)

**Time Living in the Area**	
Less than one year	136 (10%)
From 1–2 yrs	155 (12%)
From 2–5 yrs	245 (18%)
From 5–10 yrs	327 (24%)
More than 10 yrs	483 (36%)

*May not add up to 1362 due to missing data

### Time spent playing outdoors, screen time and independent mobility

Table 2 shows time spent playing outside, screen time and independent mobility as reported by children, as well as neighbourhood safety as reported by parents. Overall, 37% of children spent up to half an hour a day after school playing outdoors. More boys than girls spent two hours or more a day playing outdoors (20% vs. 12%, p < 0.001). Boys were also more likely to spend more than two hours a day on screens (i.e. TV and computer) than girls (49% vs. 36%, p < 0.001). Almost one fifth of children (18%) were never allowed to walk on their own near where they lived. Boys were more likely to have greater independent mobility than girls (53% vs. 42%, p < 0.001).

**Table 2:** Time spent playing outside, screen time, independent mobility and neighbourhood safety as reported by students and their parents

	**Girl n (%)**	**Boy n (%)**	**Total n (%)**	**Chi-Square** **P value**
**Outdoor Play Time**				
T ≤ 1/2 hour	271 (38%)	231 (36%)	502 (37%)	7.34 P < 0.001
1/2 hour < T ≤ 1 hour	165 (23%)	123 (19%)	288 (22%)	
1 hour < T ≤ 2 hours	186 (26%)	156 (25%)	342 (26%)	
T > 2 hours	83 (12%)	126 (20%)	209 (16%)	

**Screen Time**				
T ≤ 1/2 hour	105 (15%)	77 (12%)	182 (14%)	15.41 P < 0.001
1/2 hour < T ≤ 1 hour	105 (15%)	95 (15%)	200 (15%)	
1 hour < T ≤ 2 hours	236 (34%)	152 (24%)	388 (29%)	
2 hours < T ≤ 3 hours	135 (19%)	141 (22%)	276 (21%)	
T > 3 hours	120 (17%)	171 (27%)	291 (22%)	

**Independence**				
Mostly	283 (42%)	316 (53%)	599 (47%)	17.15 P < 0.001
Sometimes	244 (37%)	202 (34%)	446 (35%)	
Never	142 (21%)	84 (14%)	226 (18%)	

**Neighbourhood Safety reported by parents**				
Safe	390 (57%)	375 (61%)	765 (59%)	2.39 P = 0.068
Not sure or not safe	296 (43%)	239 (39%)	535 (41%)	

"Independence" is assessed by a question "are you allowed to walk on your own, near where you live" as reported by students.

"Neighbourhood Safety" is assessed by a scale from 1 to 5 of agreement with the statement "I live in a safe neighbourhood" as reported by parents.

More than half of the parents or caregivers (59%) either agreed or strongly agreed with the statement, "I live in a safe neighbourhood". There was no significant difference in relation to the perception of neighbourhood safety between boys' and girls' parents and carers. About one third (30%) of parents and carers neither agreed nor disagreed with this statement.

### Factors associated with time spent playing outdoors

On bi-variate analysis, factors that were associated with time spent playing outdoors after school included children's independent mobility, language spoken at home, employment status of parents and caregivers, length of time living in the area, neighbourhood safety and number of children (p < 0.10) (Table 3). However, factors including child's gender, school year, parent's age, educational level of parent and whether the parent lived with a partner were not found to be associated with time spent outdoors.

**Table 3:** Factors associated with time spent playing outside on bi-variate analysis

	**Time spent playing outside**		**Chi-Square** **P value**
	
	**> 0.5 hour** **N (%)**	**< 0.5 hour** **N (%)**	
**Independence**			
Mostly	427 (53%)	179 (37%)	44.58 P < 0.001
Sometimes	273 (34%)	177 (37%)	
Never	103 (13%)	124 (26%)	

**Neighbourhood Safety**			
Safe	494 (61%)	269 (56%)	3.42 P = 0.06
Not sure or not safe	320 (39%)	216 (45%)	

**Child's Gender**			
Girl	434 (52%)	271 (54%)	0.64 P = 0.43
Boy	405 (48%)	231 (46%)	

**School Year**			
Year 5	423 (50%)	265 (53%)	0.646 P = 0.42
Year 6	416 (50%)	238 (47%)	

**Language spoken at home**			
English	550 (65%)	239 (47%)	40.457 P < 0.001
Other	298 (35%)	267 (53%)	

**Career**			
Mother	668 (80%)	393 (79%)	0.781 P = 0.38
Father	153 (18%)	88 (18%)	
Other	13 (2%)	15 (35)	

**Employment Status**			
Full-time & part time	597 (71%)	311 (62%)	11.852 P < 0.001
Other	239 (29%)	188 (38%)	

**Parent Education**			
Primary and some high school	108 (11%)	77 (14%)	1.545 P = 0.214
Completed high school	227 (22%)	108 (20%)	
Technical certificate or diploma	243 (24%)	148 (27%)	
University/other tertiary degree	436 (43%)	220 (40%)	

**Living with Partner**			
Yes	602 (84%)	348 (83%)	0.35 P = 0.56
No	113 (16%)	72 (17%)	

**Time Living in Area**			
Less than one year	88 (11%)	48 (10%)	4.14 P = 0.04
From 1–2 yrs	89 (11%)	65 (13%)	
From 2–5 yrs	142 (17%)	102 (20%)	
From 5–10 yrs	190 (235)	135 (27%)	
More than 10 yrs	331 (39%)	149 (30%)	

**Number of Children**			
1	200 (24%)	136 (27%)	3.56 P = 0.06
2	396 (48%)	243 (49%)	
3	177 (21%)	97 (19%)	
4+	54 (7%)	23 (5%)	

The results of the multivariate analysis are shown in Table 4. The association between time spent playing outdoors after school and children's independent mobility retained its significance after adjusting for other confounders. Compared with children who were never allowed to walk on their own near where they lived, children who had been allowed to walk on their own sometimes or mostly were significantly more likely to spend more than half an hour a day outdoors after school with AORs of 1.74 (95% CI 1.24–2.45; P < 0.001) and 2.56 (95% CI 1.84–3.58; P < 0.001) respectively. Children who spoke English at home were also more likely to spend more time playing outdoors compared to non-English speaking children with an AOR of 1.99 (95% CI 1.54–2.56; P < 0.001). The parent being employed and neighbourhood safety were also significantly associated with children's time spent outdoors. Stratification by gender did not show any differences between boys and girls in relation to the variables that were associated with time spent playing outdoors.

**Table 4:** Factors associated with time spent playing outside using logistic regression modelling

	**Spending more than 0.5 hour playing outside per day**			
**Study variables**	**n (row %)**	Adjusted OR*	**95% CI**	**p**

**Independence**				
Never	103 (45%)	1		
Sometimes	273 (61%)	1.74	1.24–2.45	P < 0.001
Mostly	427 (71%)	2.56	1.84–3.58	P < 0.001

**Neighbourhood Safety**				
Not sure or not safe	320 (61%)	1		
Safe	494 (65%)	1.28	1.0–1.64	P = 0.05

**Employment Status**				
Other duties	239 (56%)	1		
Employed	597 (66%)	1.37	1.06–1.78	P = 0.02

**Child's Gender**				
Girl	434 (62%)	1		
Boy	405 (67%)	1.02	0.80–1.30	P = 0.90

**Language spoken at home**				
Other	298 (53%)	1		
English	550 (70%)	1.99	1.54–2.56	P < 0.001

**Time Living in the area**				
≤ 2 years	177 (61%)	1		
> 2 years	663 (63%)	0.91	0.68–1.23	P = 0.55

**Number of Children at home**				
1	200 (60%)	1		
> 1	627 (63%)	0.89	0.67–1.17	P = 0.40

* adjusted for all other variables in the table.

## Discussion

In this cross-sectional study, we found that a significant proportion of children (37%) spent less than half an hour a day playing outdoors after school and 43% of children spent more than two hours a day on screen time. These findings have important implications for health promotion in addressing outdoor play and reducing excessive screen time among children. Previous research suggests that outdoor time is positively and significantly associated with children's physical activity [4-6] and excessive screen time (more than 2 hours a day) is also found to be significantly associated with overweight and obesity [1,13,14].

In addition, this study found that children's independent mobility was a significant factor associated with their time spent playing outdoors after adjusting for other confounders including parent's perception of neighbourhood safety. Children's independent mobility was initially examined in relation to their independent mobility. In the UK, 61% of primary school children were rarely or never allowed to go outside without an adult to walk to school or for leisure. These children were twice as likely to be driven to school than to walk, after adjusting for parents' perception of safety [15]. The frequency of walking to school was also found to be dependent on child's independent mobility; in New South Wales 58% of parents to children 5- to 12-years-old never allowed their child to walk alone in their neighbourhood, and these children were 42% less likely to frequently walk to school [16]. To our knowledge this is the first time such an association between children's independent mobility and their time spent playing outdoors has been tested in a quantitative study with a large sample size.

Several studies have touched on the issue about children's independence and physical activity but they have mostly explored it from the perspective of the parents. That is, these studies have considered why parents may restrict independence but have not thoroughly explored how a child's independent mobility influences his/her physical activity levels. For instance, some studies have explored the factors which influence parents' decisions regarding the selection of appropriate play areas and travel methods [7,17-21] and these decisions would have a direct influence on the independent mobility of children. Prezza et al argue that a parent's psychosocial characteristics (e.g. their fear of crime, perceived risk of traffic, sense of community etc.) influence the amount of independence that they allow their children [17].

Other studies have explored the decline in children's unsupervised play and the concurrent increase in organised games [22,23]. These studies suggest that the increased participation in organised games can be attributed to, at least partly, parents' views that this will keep children safer than unstructured play. As Evans points out "traditional playgrounds – the backyards and streets – are no longer play-friendly... [and] most parents are reluctant to allow children to play unless they are easily visible or an adult is present." [22] Organised games appeal to this reluctance as adults are able to direct play and ensure safety.

Similarly, the changing nature of play spaces has been found to make parents more wary of allowing children to play unsupervised [24-26]. In fact, both Hillman and Burdette and Whitaker argue that in trying to protect children we may actually be having detrimental effects on their social and emotional development [25,26].

Caution needs to be taken when interpreting these findings as a result of the cross-sectional nature of this study and its reliance on measures that were self-reported by children and parents. The weakness of the study design and measurement errors (e.g. the possibility that the students may have over- or underestimated their time spent outdoors) may have weakened the conclusions generated from this study. The generalisation of our study results is also limited due to selection of the study population. However, this study reflects well the multicultural profile of the population in the study area and does provide some evidence linking children's independent mobility and time spent playing outdoors.

## Conclusion

Our findings of this relationship have important implications for promoting physical activity among children. They suggest that environments that promote greater independent mobility in children aged 10–12 may increase their physical activity levels and hence reduce their risk of overweight and obesity. Health promotion programs, therefore, need to acknowledge the independent mobility of children and its relation with children's outdoor play. Program components need to address parents' concerns to allow their children to play and walk around their places. The findings also suggest that children's dependence should be considered in research and evaluation into children's play and physical activity. Equally, the reasons why children from a non-English speaking background were less likely to spend more time playing outdoors should be further explored.

## Competing interests

The authors declare that they have no competing interests.

## Authors' contributions

LMW conceived the idea of this study and undertook data analysis and interpretation and wrote the original draft. JK, DM and CR contributed to writing up this manuscript. All authors have read and approved the final manuscript.
